# Maize harvester gearbox design modification for improved fatigue life

**DOI:** 10.1038/s41598-022-19982-z

**Published:** 2022-09-16

**Authors:** Ji-Tae Kim, Ho-Seop Lee, Jung-Ho Park, Jea-Keun Woo, Il-Su Choi, Young-Keun Kim, Seung-Je Cho, Chang-Sub Ha, Young-Jun Park

**Affiliations:** 1 Department of Biosystems Engineering, Seoul National University, Seoul, 08826 Republic of Korea; 2 Convergence Major in Global Smart Farm, Seoul National University, Seoul, 08826 Republic of Korea; 3 Research Institute of Agriculture and Life Sciences, Seoul National University, Seoul, 08826 Republic of Korea; 4 National Institute of Agricultural Sciences, Rural Development Administration, Jeonju, 54875 Republic of Korea; 5 Department of Bioindustrial Machinery Engineering, Jeonbuk National University, Jeonju, 54896 Republic of Korea; 6 Smart Agricultural Machinery R&D Group, Korea Institute of Industrial Technology, Gimje, 54325 Republic of Korea; 7 Dooroo Machinery & Trading Co, Asan, 31420 Republic of Korea

**Keywords:** Engineering, Physics

## Abstract

The gearbox has the advantage of being able to change the torque and rotational speed according to the gear ratio and has high power transmission efficiency by transmitting power through the contact of the gear pair. When evaluating the strength and fatigue life of a gearbox using a design load or an equivalent load, there is a possibility that the results will be very different from the actual ones. Therefore, in this study, the load duration distribution (LDD) constructed based on the actual workload was used to evaluate the strength and fatigue life of the gearbox reliably. As a result of evaluating the strength and fatigue life of the gearbox using LDD, it was confirmed that the existing gearbox did not satisfy the target lifespan in the operating environment. Therefore, the reasons for these results were analyzed, and design modification was performed based on the analyzed results. As a result of design modification, shaft deflection decreased by rearrangement of the bearings, from an overhung type to a straddle type, thereby improving the fatigue life of gears and bearings. Finally, the load distribution acting on the gear tooth surface was improved through micro-geometry modification of the gears.

## Introduction

A gear is a machine element in power transmission that is widely used in various fields^1^. A gearbox is a power transmission system that consists of gears, shafts, bearings, and housings; and the power input to the shaft is transmitted to the driven gear (herein, gear) through the driving gear (herein, pinion). Additionally, when power is transmitted using a gear-pair, since the gear ratio changes the rotation speed and torque, the advantage of controlling the rotation speed and torque by changing the gear ratio arises. The performance of a gearbox can be evaluated by parameters such as fatigue life, noise, vibration, and power transmission efficiency. In the case of fatigue life, since it determines whether the gearbox operates or not, it needs to reliably predict and evaluate the life of the gearbox^2^.

For reliably predicting and evaluating the performance of the gearbox, it is necessary to define the load acting on the gearbox accurately. The load magnitude of the load acting on the gearbox, duration under the load, and the fluctuation range of the load are determined according to the purpose and environment of use of the gearbox. However, it is challenging to numerically define the load acting on the gearbox. Therefore, many researchers used the cumulative fatigue damage theory based on the Palmgren–Miner rule and predicted and evaluated the gearbox performance under equivalent load conditions using the concept of averages^3,4^. While using the equivalent load in evaluating the gearbox’s performance can shorten the calculation time, there is the disadvantage of being unable to consider the effect of the load fluctuation and peak load acting on the gearbox. Additionally, the fatigue damage exponent used to derive the equivalent load is a value that varies depending on the failure mode of each element constituting the gearbox. At the design stage, the fatigue damage exponent cannot be accurately determined because the key failure modes of the gearbox are not available in advance^5–7^.

Dong et al.^8^ conducted a study on the effect of fluctuating wind speed on the gear contact fatigue of a wind turbine gearbox. The gear contact fatigue was analyzed using a total of 11 different wind speeds—available in literature—for implementing the wind speed fluctuations. However, since this analysis did not reflect the practical environment wherein the wind turbine gearbox is actually used, there was a limit to the reliability of the analysis results. Patel and Joshi^9^ performed a design and fatigue analysis of the gearbox carrier and confirmed that its fatigue life changed depending on its material and shape. However, the analysis suffered from the same limitation as that of the previous study, along with the additional limitation of being performed under only one load condition. Du et al.^10^ conducted a study to predict the fatigue life of the gearbox of a tracked vehicle using a running simulation test. The environment wherein the gearbox operated was simulated, and the load acting on the gearbox was derived using the simulation results. Additionally, the fatigue life of the gearbox was evaluated using the derived load. However, since the derived load was not validated, there was a limit to the reliability of the simulation results. Kim et al.^11^ built a transmission simulation model of a tractor using commercial software and developed a model that could evaluate the fatigue life of spiral bevel gears. Additionally, the load generated in the operating environment was measured, and the fatigue life of the spiral gear was predicted by constructing a load spectrum based on the measured data. The load duration distribution (LDD) method was intended for predicting the performance of the gears and bearings^12^; this study incorrectly predicted their performance with the load spectrum using the rainflow-counting algorithm. Similarly, in most studies conducted in various fields that predicted and evaluated gearbox performance, the definition of the operating environment was insufficient. Wang et al.^13^ conducted research on the design, modeling, and analysis of offshore wind turbine drivetrains. An iterative design procedure was presented to minimize the weight and volume when designing the drivetrain of wind turbine, and the model was validated by comparing the designed multibody simulation model with the previously developed model. However, there is a limitation in that the design load rather than the actual environment load was used when designing and validating the drivetrain of wind turbine. Yoo et al.^14^ developed a simulation model of the wind turbine gearbox to confirm the performance of the planetary gear set to which the flexible pin was applied. The simulation was performed using commercial software. As a result of the study, it was confirmed that load sharing and load distribution among planet gears were improved when flexible pins were applied to the planetary gear set. However, there is a limitation in that the environment in which the wind turbine gearbox is operated was assumed in performing the performance of the planetary gear set.

To solve this problem, Kim et al.^15^ performed an actual harvesting operation using a maize harvester developed by Kang et al.^16^. A sensor that could measure torque and rotational speed was attached to the tractor power take-off (PTO), and the actual workload generated during maize harvesting was measured using the sensor. Additionally, using the measured real workload, a load duration distribution that could evaluate machine elements that transmitted or supported a load through contact, such as gearbox components like gears and bearings, was constructed.

In this study, the gearbox simulation model of the maize harvester introduced by Kang et al.^16^ was developed using the commercial software Romax Nexus^17^. Additionally, the strength and fatigue life of the gears and bearings in the gearbox were evaluated using the gearbox model and the LDD constructed by Kim et al.^15^. The evaluation revealed that the gearbox did not satisfy the target fatigue life of the maize harvester; the target fatigue life was satisfied by modifying the bearing arrangement and shaft length, which are design variables of the gearbox. Finally, by performing gear micro-geometry modification, the load distribution acting on the gear tooth surface was improved.

## Methods

All methods were carried out in accordance with relevant guidelines and regulations and obtained permission from the Research Institute of Agriculture and Life Science, Rural Development Administration for collecting maize.

### Load duration distribution

Fatigue failure occurs when machine elements are subjected to fluctuating loads of varying magnitudes for many cycles. To check the safety of machine elements against fatigue failure, the external load acting on the element should be measured under the actual load condition. The load should then be processed according to the results of the safety evaluation. Among gearbox components, machine elements that transmit or support loads through contacts, such as gears and bearings, can constitute a load spectrum with the load magnitude, speed, and duration under the load^18^.

**Figure 1:**
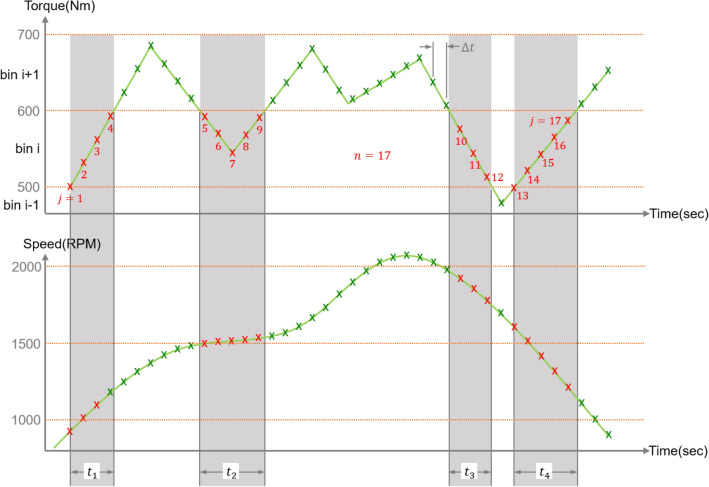
Sample data for explaining LDD method.

Figure 1 shows the sample data used for explaining the LDD method. The interval is divided into arbitrary load bins after the minimum and maximum values are checked in the measured torque data. In the sample data, the interval between the minimum and maximum torques of 500 and 670 Nm, respectively, is divided into two sections with an interval of 100 Nm. The magnitude of the load in the *i*th section of the sample data is obtained as an average of torque values from 500 to 600 Nm. The time data of the *i*th section is ${t}_{1}+{t}_{2}+{t}_{3}$, which is the total time of exposure to the torque. Finally, the speed data of the *i*th section is obtained as an arithmetic average of the rotational speeds belonging to the time data corresponding to the section. In LDD, the magnitude, duration, and speed of the load are expressed through the following equations:

1 $${T}_{i}= \frac{\sum_{j=1}^{n}{T}_{i,j}}{n},$$

2 $${t}_{i}=\Delta t\cdot n,$$

3 $${\omega }_{i}= \frac{\sum_{j=1}^{n}{\omega }_{i,j}}{n},$$

where $i$ is the bin number, ${T}_{i}$ is the *i*th average torque in bin, ${T}_{i,j}$ is the *i*th *j*th torque in bin, n is the *i*th data in the bin, $\Delta t$ is the time interval of the measurement data, ${\omega }_{i}$ is the *i*th average speed of the bin, and ${\omega }_{i,j}$ is the $i\mathrm{th}$ is the speed of the bin. Table 1 shows the details of the LDD method Kim et al.^15^ used for maize harvesting.

**Table 1:** LDD for gear rating of maize harvester gearbox^15^.

Load level	Torque (Nm)	Speed (RPM)	Duration (h)	Frequency (%)
1	97.30	539.81	423.36	8.82
2	105.12	539.70	1785.60	37.20
3	114.65	539.19	1464.00	30.50
4	124.24	538.81	723.36	15.07
5	134.15	538.49	310.56	6.47
6	143.40	538.15	79.68	1.66
7	153.49	538.11	11.04	0.23
8	162.91	537.81	2.40	0.05
Sum			4800.0	100.0

### Maize harvester gearbox model

**Figure 2:**
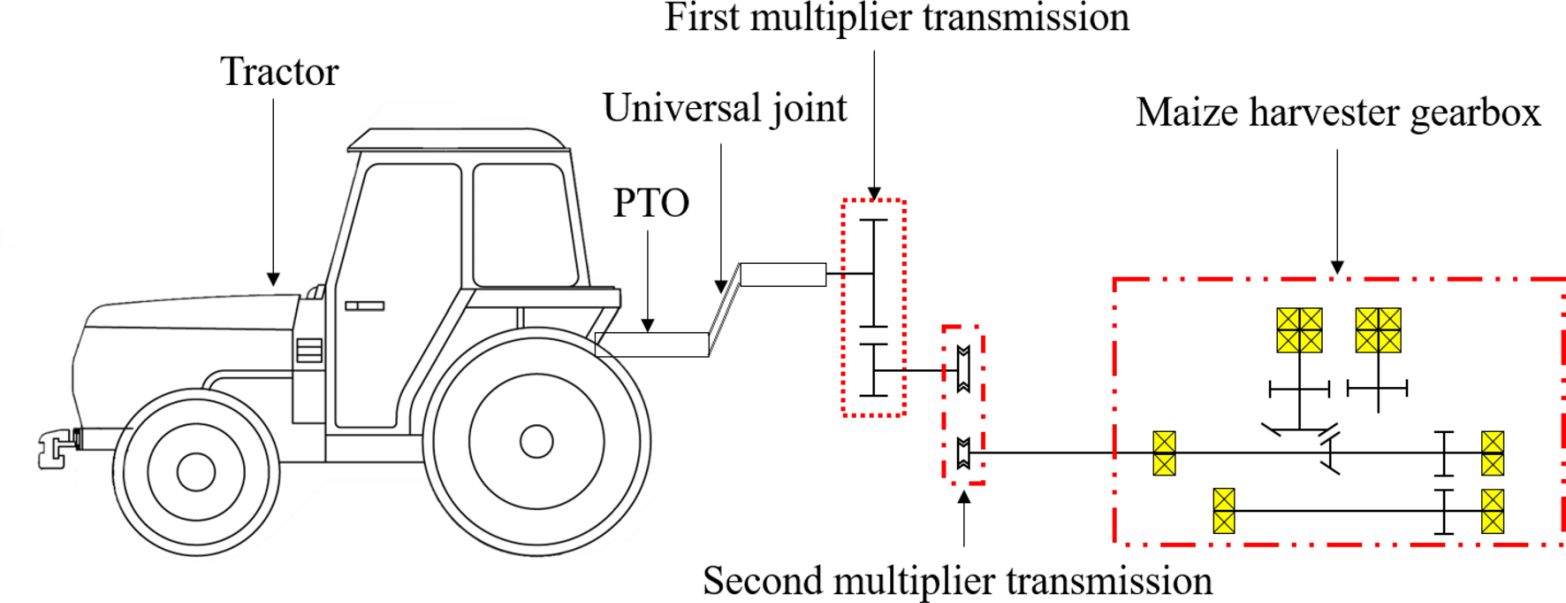
Configuration of power transmission system for maize harvester^15^.

The maize harvester consists of a maize harvester gearbox, which is used for harvesting, wherein maize stalks are transferred and peeled; the first multiplier transmission (gear ratio: 0.645), which receives power directly from the tractor PTO; and the second multiplier transmission (belt-pulley ratio: 0.714), which transmits the power of the first multiplier transmission (gear ratio: 0.645) to the maize harvester gearbox. Figure 2 shows the power transmission from the tractor PTO to the maize harvester gearbox.

**Figure 3:**
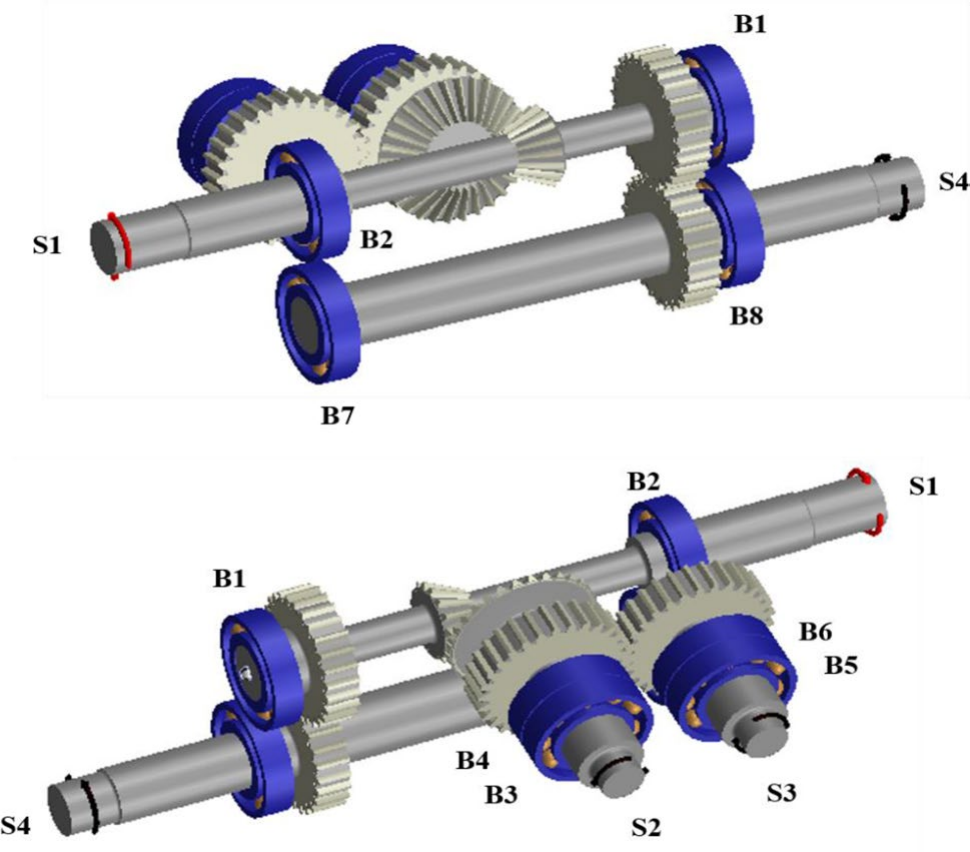
Gearbox simulation model of maize harvester.

Figure 3 shows the simulation model of the maize harvester gearbox that was developed using Romax Nexus^17^. S1, the input shaft of the maize harvester gearbox, transmits power to S2 and S3, the output shaft of the detach part, and S4, the output shaft of the transport part. A bevel gear set (BGS), a machine element that can transmit power vertically, is used between S1 and S2 to transmit power to S2 and S3, which are perpendicular to S1. Additionally, the power transmitted to S2 through the BGS is transmitted to S3 through the spur gear set (SGS) 1, which is a parallel shaft power transmission element. Finally, SGS 2 is used between S1 and S4 to transmit power to the transfer unit.

In the gearbox model, the spur and bevel gears are defined with non-linear contact stiffness and represented by macro-geometric parameters (modules, number of teeth, center distance, pressure angle, face width, among others). The gear mesh misalignment and non-linear tooth stiffness were considered for contact analysis of the gear. Since the gear mesh force is influenced by the contact position of the tooth flank, we performed modeling considered and analyzed all the gear meshing points, load distributions, and boundary conditions. To analyze the contact of the gear, the slice model, assuming that each slice is operating as a spur gear and independent, was used. The non-linear stiffness model of rolling element bearing was defined as internal detail parameters (curvature of the raceways, internal clearance, roller profile, etc.). Shafts were also modeled as flexible 1D beam elements^17^. The gear specifications used in the maize harvester gearbox are shown in Tables 2 and 3, and FAG 6207 was used for all rolling bearings.

**Table 2:** Specification of bevel gear set.

Items	Bevel gear set	
	Pinion	Gear
Module (mm)	3	
Pressure angle (°)	20	
Outer pitch diameter (mm)	45	90
Number of teeth	15	30
Net face width (mm)	25.8	
Shaft angle (°)	90	
Material	SCM415	
Quality	11 (AGMA)

**Table 3:** Specification of spur gear sets 1 and 2.

Items	Spur gear set 1		Spur gear set 2	
	Gear	Pinion	Gear	Pinion
Module (mm)	3		3	
Pressure angle (°)	20		20	
Center distance (mm)	87		78	
Number of teeth	29		26	
Tip diameter (mm)	93	92.808	84	83.808
Pitch circle diameter (mm)	87		78	
Root diameter (mm)	79.500	79.308	70.500	70.308
Face width (mm)	20		20	
Material	SCM415			
Quality	1 (ISO)			

## Results and discussion

### Gearbox evaluation using a simulation model and actual load data

In this study, the gear rating and bearing fatigue life were evaluated using the LDD generated based on the developed simulation model of the maize harvester gearbox and the actual workload measured during maize harvesting. The ratings for the spur gear and bevel gear were given based on ISO 6336^6^ and ISO 10300^19^, respectively. Additionally, the fatigue life of the bearings was evaluated using ISO 281^20^. Table 4 shows the rating results for the gear simulation.

**Table 4:** Gear safety factors of maize harvester gearbox using LDD.

Gear	Safety factor	
	For contact stress	For bending stress
BGS, pinion	1.25	2.71
BGS, gear	1.30	3.17
SGS 1, pinion	1.00	2.41
SGS 1, gear	1.00	2.39
SGS 2, pinion	3.36	18.68
SGS 2, gear	3.35	18.44

The gear rating results in Table 4 revealed the gear with the highest failure probability and that the failure mode for it was gear surface pitting caused by the contact stress of SGS 1 (pinion and gear). Therefore, to confirm that the face load distribution had a dominant influence on the safety factor for the contact stress, the face load distribution was analyzed using the finite element model and non-linear contact model of Romax Nexus^17,19^. The finite element model and non-linear contact model analyzed the face load distribution using the following four theories and calculated the face load factor (${K}_{H\beta }$) using the analysis results^17^:

1. Bending based on Mindlin plate theory;
2. Compression based on Timoshenko beam theory;
3. Root rotation based on an empirical theory;
4. Root shear based on an empirical theory.

**Figure 4:**
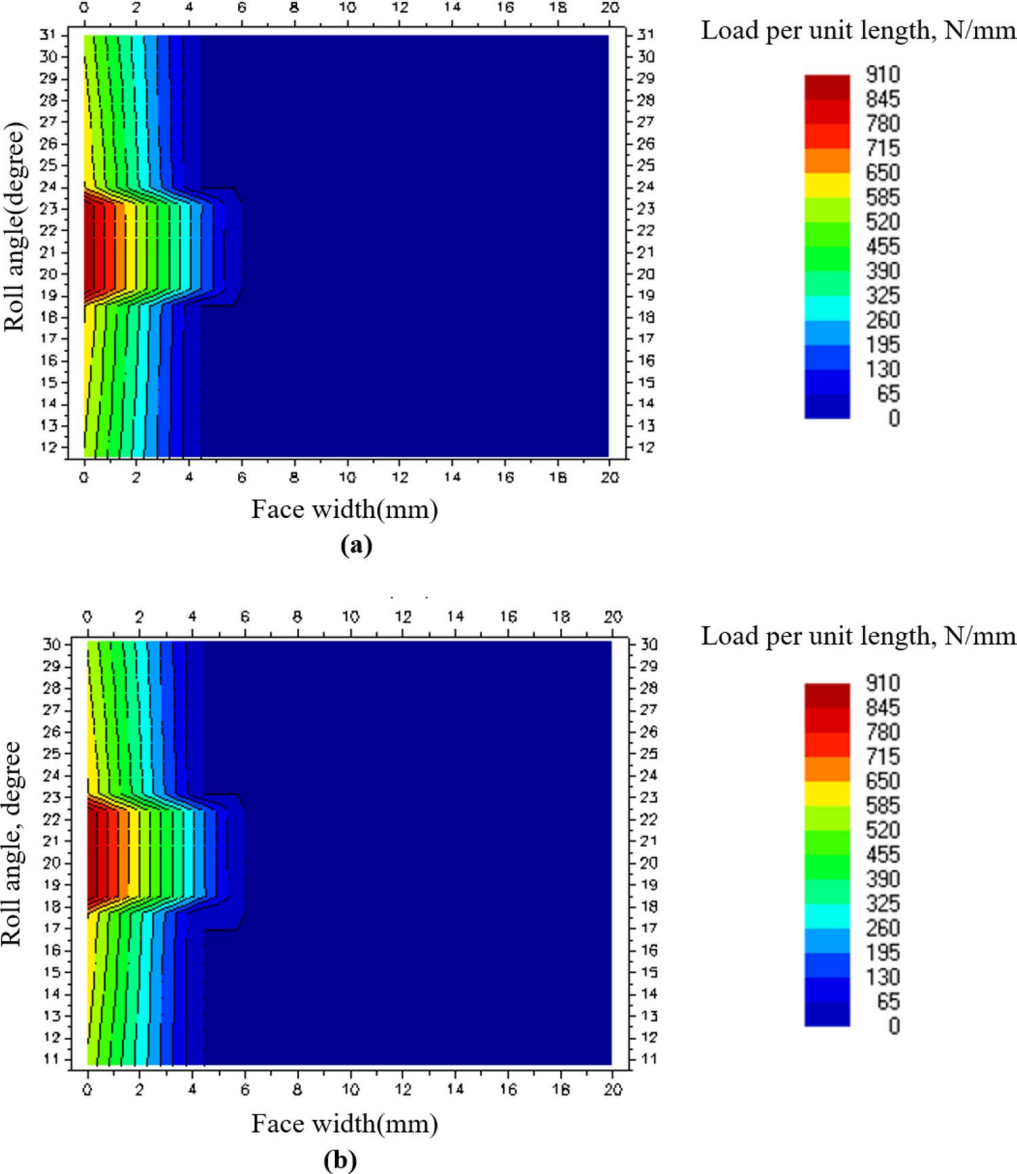
Face load distribution of SGS 1 on load level 8: (**a**) Pinion contact pattern and (**b**) gear contact pattern.

Table 5 shows the maximum load per unit length and face load factor of SGS 1, and Fig. 4 shows the face load distribution at load level 8 of SGS 1. From Fig. 4, it was confirmed that the contact pattern of SGS 1 was extremely skewed to the left. As a result, the tooth surface area that transmitted the load was reduced, and it was confirmed that the safety factor for the contact stress was low owing to the induced high contact stress.

**Table 5:** Face load factor of SGS 1 according to load level in LDD.

Load level	Maximum load per unit length (N/mm)	${K}_{H\beta }$
1	335	8.36
2	395	8.04
3	491	7.66
4	575	7.40
5	664	7.18
6	740	7.03
7	832	6.82
8	910	6.62

Tables 6 and 7 show the fatigue life evaluation results of radial and axial loads of all the bearings acting on each bearing at load level 8. Table 6 confirms that the bearings B3 and B4 located in S2 did not satisfy the target fatigue life of 4800 h for the maize harvester gearbox, and Table 7 confirms that the load acting on these bearings is very large.

**Table 6:** Lifetime of bearings in maize harvester gearbox in LDD.

Rolling bearing		Lifetime (h)
S1	B1	32,777
	B2	1.3 × 10^5^
S2	B3	2503
	B4	622
S3	B5	4.7 × 10^5^
	B6	58,963
S4	B7	8.6 × 10^9^
	B8	1.2 × 10^7^

**Table 7:** Reaction force of bearings on load level 8 in LDD.

Rolling bearing	Radial force (N)	Axial force (N)
B1	3732.2	1942.1
B2	2968.9	916.5
B3	10,931.1	5606.4
B4	19,650.3	7657.5
B5	19,67.3	625.9
B6	4546.3	625.9
B7	72.3	0.4
B8	646.8	0.4

### Design modification of maize harvester gearbox

From the results of the gearbox simulation, it was confirmed that the weak parts of the maize harvester gearbox were B3 and B4, located at SGS 1 and S2, respectively. In this study, it was determined that the cause of the occurrence of the weak part of the gearbox was as follows.

1. The arrangement of B3 and B4 in S2 as an overhung type was unfavorable to moment support.
2. The moment was generated in S2 due to the gear mesh force of BGS and SGS 1 that led to the deflection of S2.
3. Gear mesh misalignment occurred in SGS 1 owing to the deflection of S2.
4. The Increased ${K}_{H\beta }$ and reduced safety factor for contact stress was due to gear mesh misalignment.

**Figure 5:**
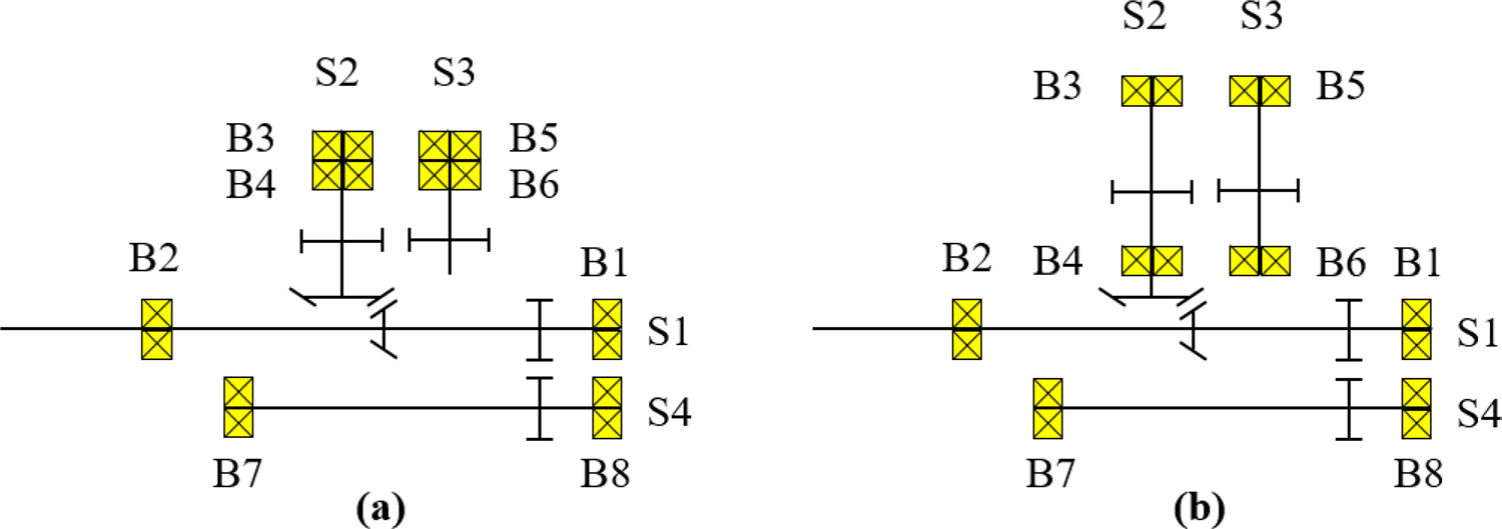
Change in bearing arrangement according to design modification of gearbox: (**a**) Before and (**b**) after design modification.

**Figure 6:**
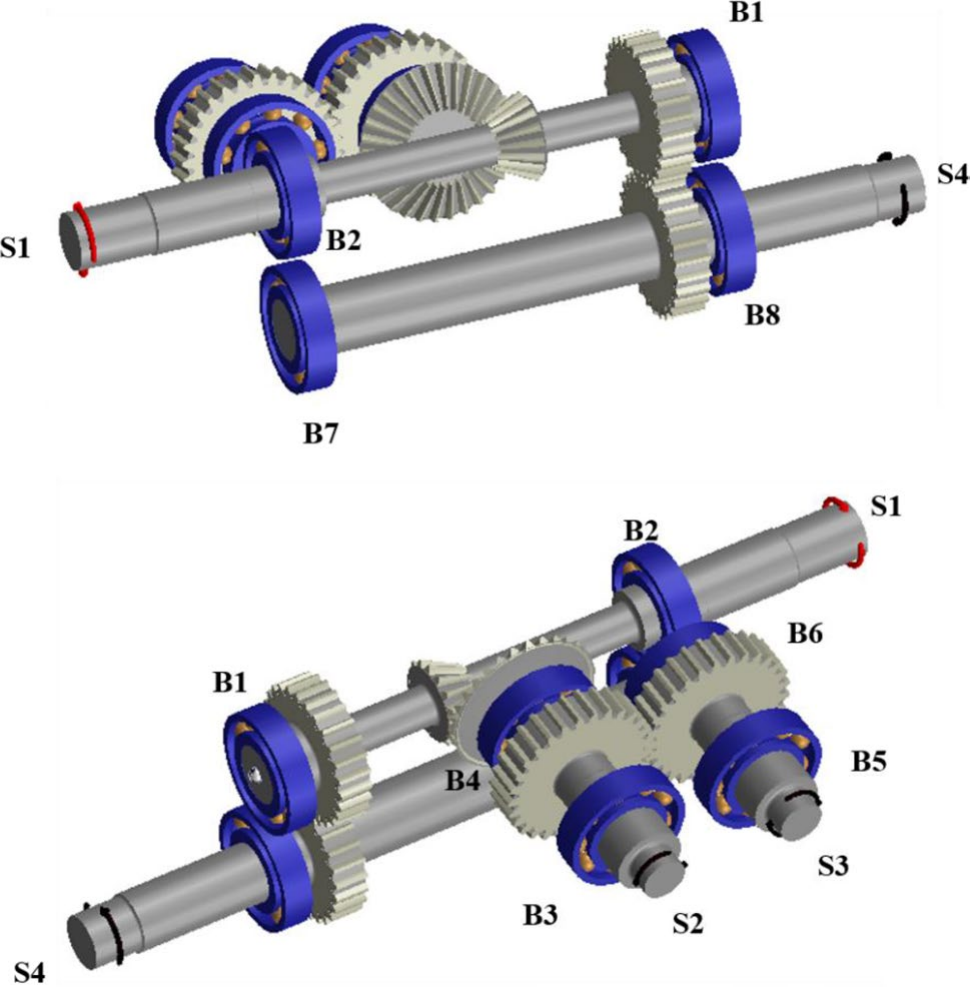
Modified gearbox simulation model of maize harvester.

To solve the above problem, the shaft length between the bevel gear and the spur gear of S2 was increased by 20 mm, as shown in Fig. 5. Additionally, by positioning B4 between the bevel gear and the spur gear, B3 and B4 were arranged like a straddle, an arrangement advantageous for moment support. Finally, the shaft length of S3 was increased by 20 mm to position B6 at the top together with B4. Figure 5 shows the bearing arrangement before and after the design modification, and Fig. 6 shows the modified simulation model.

As performed for the model before design modification, the gear rating and bearing fatigue life of the design-modified simulation model were evaluated using LDD, and the results are shown in Tables 8, 9 and 11.

**Table 8:** Gear safety factor of modified gearbox in LDD.

Gear	Safety factor	
	For contact stress	For bending stress
BGS, pinion	1.25	2.71
BGS, gear	1.30	3.17
SGS 1, pinion	1.95	5.99
SGS 1, gear	1.95	5.93
SGS 2, pinion	3.36	18.68
SGS 2, gear	3.35	18.44

**Table 9:** Face load factor of SGS 1 in modified gearbox according to load level in LDD.

Load level	Maximum load per unit length (N/mm)	${K}_{H\beta }$
1	85	2.10
2	102	2.04
3	129	1.98
4	154	1.93
5	180	1.90
6	202	1.88
7	230	1.84
8	254	1.80

**Figure 7:**
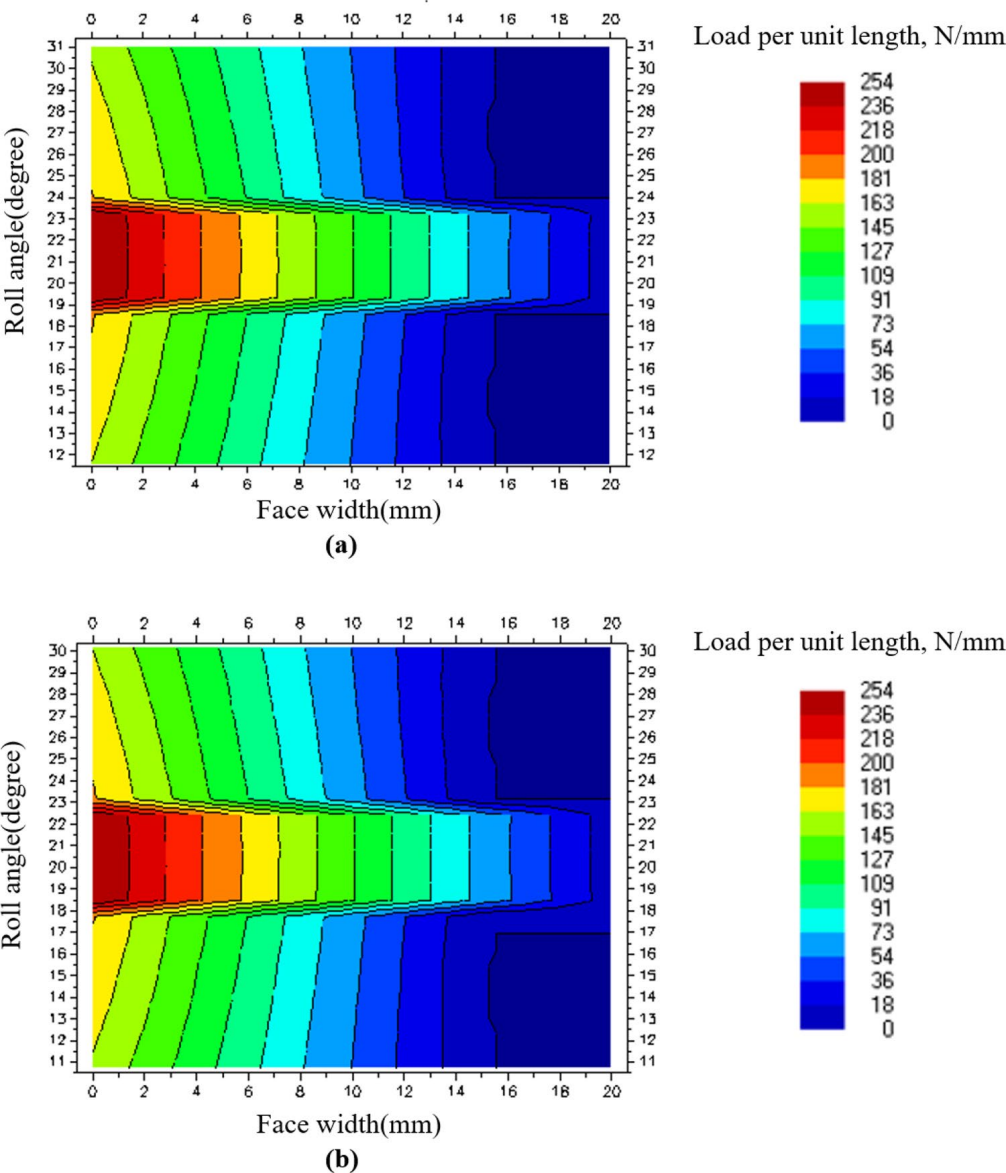
Face load distribution of SGS 1 on load level 8 for modified gearbox: (**a**) Pinion contact pattern and (**b**) gear contact pattern.

As shown in Tables 8 and 9, it was confirmed that the safety factor for the contact stress of SGS 1, a weak component of the existing gearbox, increased by approximately 1.9 times owing to the design modification. According to the load level, the maximum load per unit length decreased by 3.74 times on average, and the ${K}_{H\beta }$ decreased by 3.82 times on average. As shown in Fig. 7, the decrease in the safety factor for the contact stress and maximum load per unit length was assumed to be due to the relatively even distribution of the contact pattern of SGS 1 compared to that of the existing gearbox. Also, as shown in Tables 10 and 11, the load applied to B3 and B4 was significantly reduced through the design modification. Accordingly, the lifespans of B3 and B4, which did not meet the target fatigue life in the existing gearbox, were $1.6x{10}^{8}$ and 8672 h, respectively, confirming that the target fatigue life was satisfied.

**Table 10:** Bearing lifetime of modified gearbox in LDD.

Rolling bearing		Lifetime (h)
S1	B1	32,777
	B2	1.3 × 10^5^
S2	B3	1.6 × 10^8^
	B4	8672
S3	B5	7.8 × 10^5^
	B6	1.5 × 10^6^
S4	B7	8.6 × 10^9^
	B8	1.2 × 10^7^

**Table 11:** Bearing reaction forces of modified gearbox on load level 8 in LDD.

Rolling bearing	Radial force (N)	Axial force (N)
B1	3732.2	1942.1
B2	2968.8	916.6
B3	362.5	1.2 × 10^–2^
B4	9074.9	2051.0
B5	910.8	3.2 × 10^–4^
B6	1668.4	3.2 × 10^–4^
B7	72.3	0.4
B8	646.8	0.4

### Micro-geometry modification of SGS 1 in maize harvester gearbox

**Figure 8:**
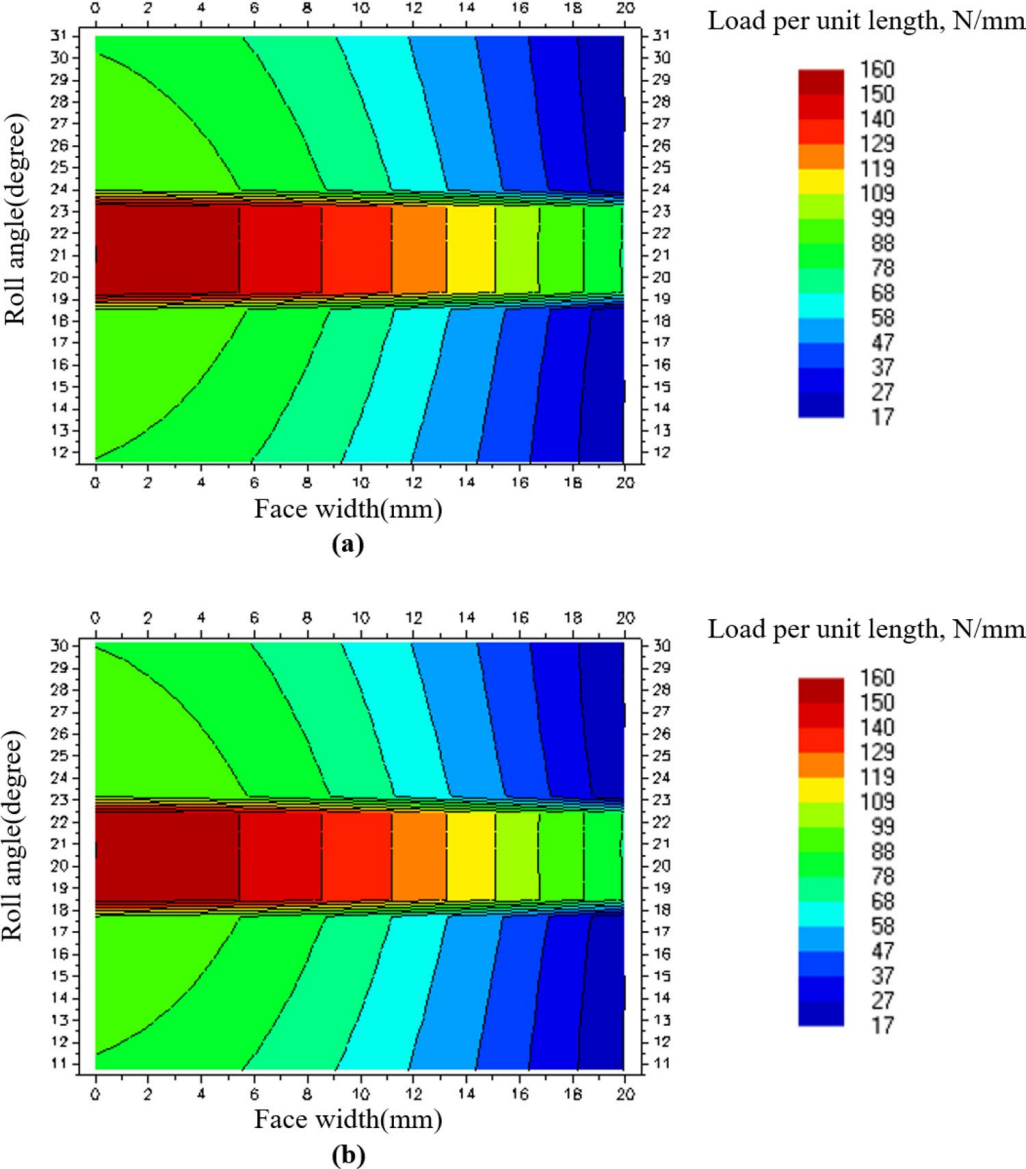
Face load distribution of SGS 1 on load level 8 after micro-geometry modification: (**a**) Pinion contact pattern and (**b**) gear contact pattern.

Although the safety factor of SGS 1 increased through the design modification of the gearbox, since the face load distribution acting on SGS 1 was still skewed to the left of gear tooth surface, it caused high contact stress and shortened the fatigue life of the gear^21^. Therefore, in this study, we performed a micro-geometry modification of the gear on SGS 1 to improve the face load distribution. This modification was performed with a lead crown and lead slope, and a parameter study was performed for a total of 121 cases, under which the crown was increased by 1 μm from 0 to 10 μm and the slope was increased by 2 μm from 0 to 20 μm. The parameter study was performed to calculate the ${K}_{H\beta }$ at each of the 8 load levels and derive the combination of the lead crown and lead slope with the smallest sum of the ${K}_{H\beta }$ values. Tables 12 and 13 show the results of the strength evaluation of the gears after the micro-geometry modification. Figure 8 shows the face load distribution at load level 8 of SGS 1, which was subjected to the micro-geometry modification.

**Table 12:** Gear safety factor of modified gearbox in LDD after micro-geometry modification.

Gear	Safety factor	
	For contact stress	For bending stress
BGS, pinion	1.25	2.71
BGS, gear	1.30	3.17
SGS 1, pinion	2.55	8.06
SGS 1, gear	2.55	7.96
SGS 2, pinion	2.92	15.78
SGS 2, gear	2.91	15.59

**Table 13:** Face load factor of SGS 1 in modified gearbox after micro-geometry modification according to load level in LDD.

Load level	Maximum load per unit length (N/mm)	${K}_{H\beta }$
1	44	1.37
2	49	1.21
3	61	1.10
4	74	1.09
5	93	1.11
6	111	1.14
7	136	1.2
8	160	1.24

The micro-geometry design modification increased the safety factor for the contact stress of SGS 1, which was the weak component of the initial design model, by about 2.55 times. Additionally, the maximum load per unit length according to the load level decreased by 7.14 times on average compared to that of the initial design model, and the ${K}_{H\beta }$ decreased by 6.27 times on average compared to that of the initial design model.

## Conclusions

The purpose of this study is to evaluate the previously developed maize harvester gearbox based on the simulation model and the actual workload. This gearbox was modeled using Romax Nexus, and the actual workload was based on pre-existing research results. The evaluation revealed that the previously developed gearbox did not satisfy the target fatigue life. This was attributed to the shaft deflection that occurred due to the bearing misarrangement and gear mesh misalignment from the internal clearance of bearing and uneven load distribution of the gear tooth surface. The required target fatigue life of the maize harvester was satisfied by calculating the shaft deflection, gear mesh misalignment, and uneven load distribution of the gear teeth.

1. The strength and fatigue life analyses of the gears and bearings in the gearbox of a maize harvester were performed using the maize harvest LDD and simulation model. The target fatigue life of the gearbox was not satisfied in B3 and B4 of S2, and the safety factor for the contact stress of SGS 1 was derived as 1.00, confirming that the gearbox required improvement.
2. Since B3 and B4 of the existing gearbox were arranged overhung over S2, a moment was generated by the gear of BGS located in S2 and the pinion of SGS 1. It was determined that deflection occurred in S2 owing to the moment, which resulted in a shortened bearing life and increased mesh misalignment for SGS 1. To solve this problem, design modification was performed to change the overhung arrangement of B3 and B4 to a straddle one. After the modification, both B3 and B4 satisfied the target fatigue life of the gearbox for the maize harvester, and it was confirmed that the safety factor for the contact stress of SGS 1 increased to an average of 1. 9. Furthermore, for the face load distribution of SGS 1, the maximum load per unit length decreased by an average of 3.77 times. However, the face load factor, which indicated the face load distribution, was as large as 1.8–2.1, confirming that further improvement was required.
3. A micro-geometry modification was performed to improve the face load distribution of SGS 1. The modification was performed on the lead crown and lead slope, and the smallest sum of the face load factors was derived at all the load levels through a parameter study. As a result, the maximum load per unit length of SGS 1 was reduced by approximately 7.14 times compared to that of the existing SGS 1, and the face load factor was found to be 1.0–1.3, which decreased by about 6.27 times on average.
4. Finally, for performing the gear strength and bearing life evaluations for the gearbox, (1) a high-precision simulation model that could accurately simulate the actual gearbox and (2) an actual load-based LDD were essential. (3) It was confirmed that the gearbox should be evaluated and design modifications should be applied, based on (1) and (2).

## Data Availability

The datasets during and/or analyzed during the current study available from the corresponding author on reasonable request.

## References

[1] Goch G (2003). Gear metrology. CIRP Ann..

[2] Lewicki DG, Black JD, Savage M, Coy JJ (1986). Fatigue life analysis of a turboprop reduction gearbox. J. Mech. Des..

[3] Shin JW, Kim JO, Choi JY, Oh SH (2014). Design of 2-speed transmission for electric commercial vehicle. Int. J. Automot. Technol..

[4] Kim TJ (2019). Strength analysis of mechanical transmission using equivalent torque of plow tillage of an 82 kW-class tractor. Korean J. Agric. Sci..

[5] Park YJ, Lee GH, Song JS, Nam Y (2013). Characteristic analysis of wind turbine gearbox considering non-torque loading. J. Mech. Des..

[6] ISO 6336:2019. Calculation of load capacity of spur and helical gears.

[7] Park YJ, Kim JG, Lee GH, Shim SB (2015). Load sharing and distributed on the gear flank of wind turbine planetary gearbox. J. Mech. Sci. Technol..

[8] Dong W, Xing Y, Moan T, Gao Z (2013). Time domain-based gear contact fatigue analysis of a wind turbine drivetrain under dynamic conditions. Int. J. Fatigue.

[9] Patel MM, Joshi NB (2015). Design and fatigue analysis of epicyclic gearbox carrier. Int. J. Innov. Res. Sci. Technol..

[10] Du XJ, Zhang SR, Zhang YH (2019). Fatigue life prediction of the gear box in tracked vehicles based on running simulation tests. Strength Mater..

[11] Kim WS (2020). Fatigue life simulation of tractor spiral bevel gear according to major agricultural operations. Appl. Sci..

[12] Germanischer, L. Guideline for the certification of offshore wind turbine workplace, access and exit-dimensions. Hamburg, Germany (2010).

[13] Wang S, Nejad AR, Moan T (2020). On design, modeling, and analysis of a 10-MW medium-speed drivetrain for offshore wind turbines. Wind Energy.

[14] Yoo HG (2022). Application of flexible pin for planetary gear set of wind turbine gearbox. Sci. Rep..

[15] Kim JT (2022). Determination of design loads of maize harvester using actual working load. J. Agric. Life Sci..

[16] Kang NR (2019). Performance evaluation and design of an edible fresh corn harvesting machine. J. Drive Control.

[17] Romax Technology, User manual, Romax Nexus (2021).

[18] Niederstucke B., Anders, A., Dalhoff, P. & Grzybowski, R. Load data analysis for wind turbine gearboxes. Technical Report, Germanischer Lloyd WindEnergie GmbH, Germany, August (2003).

[19] ISO 10300:2014. Calculation of load capacity of bevel gears.

[20] ISO 281:2007. Rolling bearings-dynamic load ratings and rating life.

[21] Park YJ, Lee GH, Kim JK, Song JS (2011). Analysis of load distribution and sharing on the planetary reducer for wind turbines. J. Korean Soc. Manuf. Technol. Eng..

